# The Variation in Practice of the Living Donor Kidney Transplant Pathway in the UK: Results of a National Survey

**DOI:** 10.3389/ti.2025.15341

**Published:** 2025-11-12

**Authors:** Katie Nightingale, Josh Stephenson, Rajesh Sivaprakasam, Tim Brown, Nicholas Inston, Ahmed Hamsho, Rommel Ravanan, Michael Nicholson, Argiris Asderakis, Sarah Browne, James Hunter, Lorna P. Marson, Katie L. Connor, Mortimer Kelleher, Andrew Sutherland, William Norton, Hannah Maple, Francis Calder, Frank J. M. F. Dor, Adam Barlow, Imeshi Wijetunga, Rachel Youngs, Stuart Falconer, Victoria Boardman, Matthew Welberry Smith, Atul Bagul, Hemant Sharma, Sanjay Mehra, Zia Moinuddin, Tunde Campbell, David van Dellen, Alistair Rogers, Lisa Burnapp, Kamran Haq, James Yates, Sanjay Sinha, Shahzar Malik, Imran Saif, Paul Gibbs, Kashuf Khan, Rafique Harvitkar, Badri Shrestha, Abbas Ghazanfar, Abul Siddiky, Reza Motallebzadeh, Michael Moneke, Kailash Bhatia, Titus Augustine

**Affiliations:** 1 Department of General Surgery, Manchester Royal Infirmary, Manchester University NHS Foundation Trust, Manchester, United Kingdom; 2 Manchester Centre for Transplantation, Manchester Royal Infirmary, Manchester University NHS Foundation Trust, Manchester, United Kingdom; 3 Royal London Hospital Transplant Department, The Royal London Hospital, Barts Health NHS Trust, London, United Kingdom; 4 Regional Nephrology and Transplant Unit, Belfast City Hospital, Belfast Health and Social Care Trust, Belfast, United Kingdom; 5 Queen Elizabeth Hospital Birmingham, University Hospitals Birmingham NHS Foundation Trust, Birmingham, United Kingdom; 6 Southmead Hospital, North Bristol NHS Trust, Bristol, United Kingdom; 7 Department of Nephrology, Urology and Renal Transplantation, Royal Free Hospital, Royal Free London NHS Foundation Trust, London, United Kingdom; 8 Cardiff Transplant Unit, University Hospital of Wales, Cardiff and Vale University Health Board, Cardiff, United Kingdom; 9 University Hospital Coventry, University Hospitals Coventry and Warwickshire NHS Trust, Coventry, United Kingdom; 10 Edinburgh Transplant Unit, Royal Infirmary of Edinburgh, NHS Lothian, Edinburgh, United Kingdom; 11 West of Scotland Kidney Transplant Unit, Queen Elizabeth University Hospital, NHS Greater Glasgow and Clyde, Glasgow, United Kingdom; 12 Department of Transplant, Renal and Urology, Guy’s Hospital, Guy’s and St Thomas’ NHS Foundation Trust, London, United Kingdom; 13 Renal and Transplant Centre, Hammersmith Hospital, Imperial College Healthcare NHS Trust, London, United Kingdom; 14 St James’s University Hospital, Leeds Teaching Hospitals NHS Trust, Leeds, United Kingdom; 15 Department of Renal Medicine and Renal Transplantation, Leeds Teaching Hospitals NHS Trust, and Faculty of Biological Sciences, University of Leeds, Leeds, United Kingdom; 16 Leicester General Hospital, University Hospitals of Leicester NHS Trust, Leicester, United Kingdom; 17 Renal and Transplant Centre, Royal Liverpool University Hospital, Liverpool University Hospitals NHS Foundation Trust, Liverpool, United Kingdom; 18 Freeman Hospital, Newcastle upon Tyne Hospitals NHS Foundation Trust, Newcastle upon Tyne, United Kingdom; 19 NHS Blood and Transplant, Bristol, United Kingdom; 20 St Peter’s Hospital, Ashford and St Peter’s Hospitals NHS Foundation Trust, Chertsey, United Kingdom; 21 Renal and Transplant Unit, Queen’s Medical Centre, Nottingham University Hospitals NHS Trust, Nottingham, United Kingdom; 22 Oxford Transplant Centre, Churchill Hospital, Oxford University Hospitals NHS Foundation Trust, Oxford, United Kingdom; 23 Royal Devon and Exeter Hospital, Royal Devon University Healthcare NHS Foundation Trust, Exeter, United Kingdom; 24 Southwest Transplant Centre, Derriford Hospital, University Hospitals Plymouth NHS Trust, Plymouth, United Kingdom; 25 Wessex Kidney Centre, Queen Alexandra Hospital, Portsmouth Hospitals University NHS Trust, Portsmouth, United Kingdom; 26 Department of Renal Transplantation, Northern General Hospital, Sheffield Teaching Hospitals NHS Foundation Trust, Sheffield, United Kingdom; 27 Department of Transplantation and Dialysis Access Surgery, St George’s Hospital, St George’s University Hospitals NHS Foundation Trust, London, United Kingdom; 28 Department of Nephrology and Transplantation, Royal Free Hospital, London, United Kingdom; 29 Department of Anaesthesia and Perioperative Medicine, Manchester Royal Infirmary, Manchester University NHS Foundation Trust, Manchester, United Kingdom; 30 Division of Diabetes, Endocrinology and Gastroenterology, Faculty of Biology, Medicine and Health, University of Manchester, Manchester, United Kingdom

**Keywords:** living donor kidney transplantation, laparoscopy, donor nephrectomy, variation, perioperative care

## Abstract

Living donor kidney transplantation (LDKT) accounts for 35% of kidney transplants in the UK. The Organ Donation and Transplantation 2030 initiative underscores the necessity to enhance LDKT rates to meet growing demand. There is limited data on national variations in live donor workup pathways from initial referral to long-term follow-up. We conducted an online survey across all 23 UK transplant centres performing LDKT, covering the entire living donor pathway. We aimed to explore and highlight practice variation and identify opportunities for improvement. Responses were received from 21 centres (91.3%). Marked variation was identified in donor acceptance criteria, including age limits, body mass index thresholds, and donor evaluation timelines (6–36 weeks). Differences were also noted in multidisciplinary team processes, kidney laterality decisions, and perioperative enhanced recovery protocols. All centres used laparoscopic techniques, with hand-assisted transperitoneal nephrectomy being most common (57.1%). Donor nephrectomy and implantation were conducted sequentially in 15 (71.4%) of centres, and in parallel in six (28.6%). Variation was also seen in follow-up duration with 47.6% of centres offering lifelong follow-up. Despite excellent national outcomes, this survey highlights significant variation. Standardising key processes could streamline donor pathways, improve experiences, and support increased LDKT activity in the UK.

## Graphical Abstract

**Figure:**
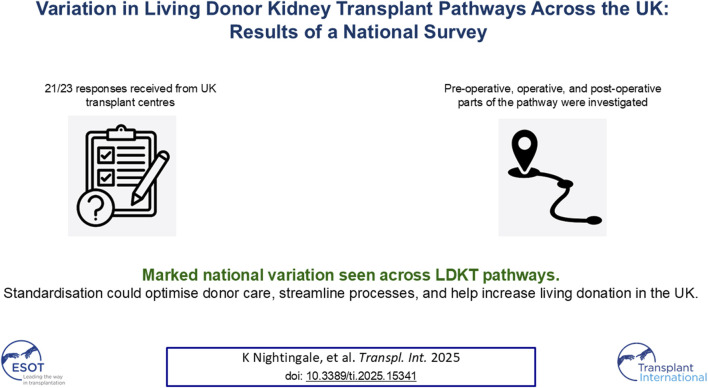

## Highlights

### What We Know

- Living donor kidney transplantation (LDKT) is vital but may be under-utilised.
- Living donors provide only 35% of UK kidney grafts in the United Kingdom (UK).
- National strategy (Organ Donation and Transplantation 2030) calls for higher uptake.

### What the Study Adds

- This national survey of 21 transplant centres in the UK highlights significant variability in the donor selection and evaluation criteria.
- Donor work-up at these centres differs beyond medical screening. Multidisciplinary-team approval steps, laterality choice, and enhanced recovery protocols are handled differently across many sites.
- Follow-up duration is inconsistent. 48% of centres guarantee lifelong monitoring; others report offering a follow-up between 3 and 24 months.

### Potential Impact

- Greater standardisation of ERAS protocols and enhanced collaboration could facilitate process optimisation and unify the donor experience to align with standards aimed at increasing LDKT activity in the UK.

## Introduction

Living donor kidney transplantation (LDKT) has, over the last 70 years, consistently proven to be the optimal form of renal replacement therapy for eligible individuals, particularly when performed pre-emptively [1, 2]. Outcomes after LDKT surpass those of deceased donor kidney transplantation, offering superior graft survival and patient longevity [3]. In the United Kingdom (UK), LDKT accounts for ∼35% of annual kidney transplants [3].

The donor pathway—from identification and evaluation, through nephrectomy and follow-up—is complex and prioritises donor safety and suitability without compromising the long-term health of the donor. The NHS Blood and Transplant (NHSBT) annual report demonstrates excellent outcomes across all 23 UK adult transplant centres [4]. Nevertheless, variation in donor evaluation and surgical pathways likely exist. The UK Transplantation 2030 strategy articulated the pressing need to increase both organ donation and transplantation rates to address a substantial unmet demand [5]. The strategy calls on all transplant centres to innovate and optimise pathways to maximise the potential for LDKT.

While perioperative variation has been studied [6], no prior study has examined variation in donor evaluation across UK centres. We conducted a national survey to explore differences in evaluation, perioperative care, and follow-up practices among MDTs performing LDKT in all UK transplant centres.

## Materials and Methods

A comprehensive online survey consisting of 65 questions was collaboratively created with contributions from clinicians, transplant coordinators, and the UK Living Donor Network, who are regularly involved in and conducting LDKT (see Supplementary Appendix S1). This survey encompassed all aspects of the donor pathway, such as evaluation timelines, discussions regarding surgical risks, criteria for donation (including age, body mass index (BMI), and co-morbidities), the use of imaging, and kidney selection for nephrectomy.

Questions pertaining to the perioperative phase included details about admission, whether surgeries were performed sequentially or concurrently, enhanced recovery after surgery (ERAS) protocols, surgical techniques, management of vascular issues, perfusion fluids, anaesthesia, fluid management, as well as post-operative care and follow-up schedules.

The survey was disseminated to transplant leads at all 23 UK centres from 1 December 2023 to 31 December 2024, with two reminders issued to those who did not respond. Each centre completed the survey after engaging in multidisciplinary discussions to reduce individual bias. Data collection was conducted in two phases: an initial questionnaire followed by a subsequent follow-up sent to all respondents to clarify and elaborate on emerging themes. The extended collection period reflects this two-phase approach; centres were requested to report their current routine practices at the time of their response, thereby reducing temporal variation.

According to the guidelines set forth by the Health Research Authority UK [7], ethical approval was not deemed necessary, and the study was registered with our local governance department [8]. The responses were analysed utilizing descriptive statistics.

## Results

21 of 23 (91.3%) centres responded. Results are grouped into pre-operative, intra-operative, and post-operative variations.

### Preoperative Evaluation

#### Evaluation in Clinic

**FIGURE 1:**
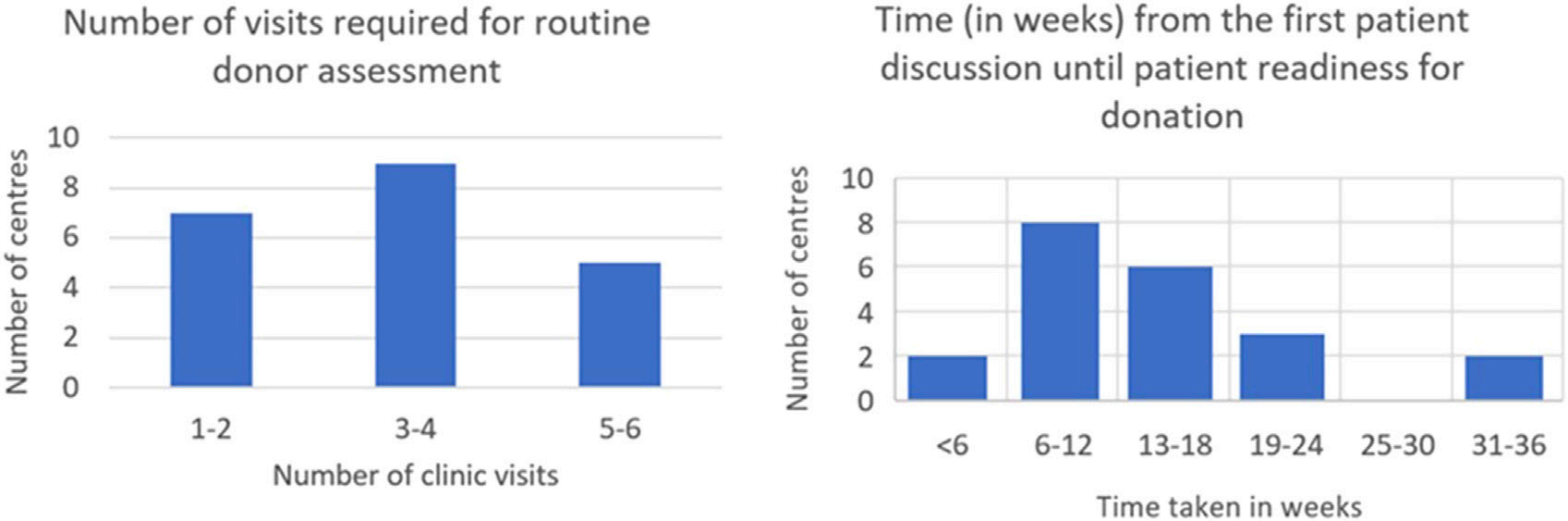
Time and visits required for donor nephrectomy assessment.

An 18-week donor turnaround time is offered by 16 (76.2%) of centres with two centres (9.5%) providing expedited pathways of under 6 weeks. The number of preoperative clinic visits required varies widely across centres (Figure 1). 16 (76.2%) centres operate distinct surgical and nephrology clinics. Five centres (23.8%) provide combined clinics, while eight centres (38.1%) include living donor MDT clinics with supplementary anaesthetic evaluations. Seven centres (33.3%) also integrate independent assessment clinics with surgical or medical assessment clinics to minimise the number of appointments and expedite donation.

#### Surgical Risk Estimates

Within the clinics, the mortality rate communicated to patients varies between 1:1500 and 1:6000. One centre (4.8%) cites a rate of 1:1500–3000; thirteen centres (61.9%) report a rate of 1:3000, three centres (14.3%) mention 1:3500, two centres (9.5%) indicate a range of 1:3000-1:4000, one centre (4.8%) describes a range of 1:3000-1:6000, and one centre (4.8%) discusses the risk as being less than 1%.

For the risk of kidney failure, the rates communicated to patients range from 1:1000 to 1:7000. Four centres (19.1%) utilise the Johns Hopkins Risk Calculator to tailor the risk assessment [9]; while the remaining centres rely on published literature. Among these 17 centres, four (23.5%) report a risk of 1:1000, one centre (5.9%) states 1:3500, another centre (5.9%) mentions 1:7000, six centres (35.3%) indicate a risk of less than 1%, two centres (11.8%) quote a risk of 1:200, and three centres (17.6%) discuss a risk that is 5–10 times greater than the current risk.

Risk information was delivered predominantly by surgeons, with some centres involving nephrologists or donor advocates.

#### Donor Selection Criteria

**FIGURE 2:**
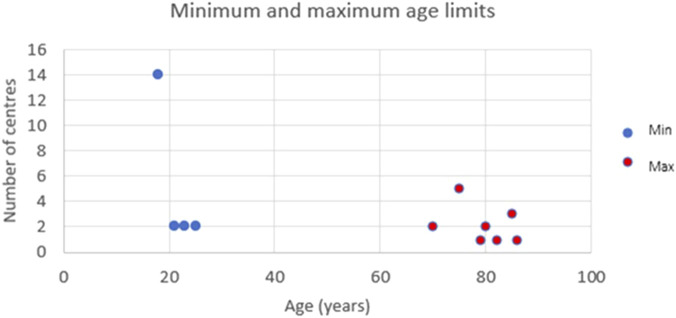
The minimum and maximum accepted age ranges per centre for donor nephrectomy.

Fifteen centres, representing 71.4%, accept donors aged 18 years or older; six centres, accounting for 28.6%, require donors to be at least 21 years old. The upper age limits vary, with two centres (9.6%) setting the limit at 70 years, while four centres (19%) have no age cutoff. One centre (4.8%) has reported accepting a 90-year-old for LDKT. (Figure 2).

Eleven centres (52.4%) accept donors with a BMI exceeding 30 kg.m^−2^, of which five centres (45.5%) impose an upper limit of 35 kg.m^−2^. Five centres (23.8%) report a minimum BMI threshold of 17–18 kg.m^−2^, whereas 16 centres (76.2%) did not have a minimum threshold.

All centres (100%) accept donors with hypertension that is managed with one medication, while fifteen centres (71.4%) accept donors on two medications. Additionally, 19 centres (90.5%) are willing to accept Jehovah’s Witnesses as donors, while two centres (9.5%) do not permit this.

#### Imaging

All centres utilise CT angiograms; additionally, two (9.5%) employ MR angiograms to outline vascular anatomy. All centres reported favouring the left kidney because of its longer vein, although anatomy, size, and function also play a role in decision-making.

### Intraoperative Differences

#### Admission

Thirteen (61.9%) centres evaluate the venous thromboembolism (VTE) risk upon admission; seven (33.3%) provide preoperative intravenous (IV) fluids; four (19%) administer pre-emptive analgesia; five (23.8%) utilise carbohydrate-loading drinks.

Ten (47.6%) centres indicate that they admit donors the day prior to surgery; ten (47.6%) admit them on the same day; one (4.8%) allows for both options. Eight (38.1%) have cross-matched blood routinely available, while thirteen (61.9%) rely on group and save.

#### Surgical Technique

All responding centres conduct laparoscopic nephrectomy, utilising one of five main techniques: 12 (57.1%) provide hand-assisted, eight (38.1%) offer totally laparoscopic, and two (9.5%) each implement hand-assisted or fully retroperitoneal technique. Robotic-assisted nephrectomy is either available or planned at 16 (76.2%) centres. The extraction incisions differ: Pfannenstiel and iliac fossa (38.1% each), supra-umbilical (28.6%), and infra-umbilical and hypochondrial (4.8% each).

#### Conduct of Surgery

15 (71.4%) centres operate sequentially; and six (28.6%) operate in parallel. 18 (85.7%) use separate surgical teams for donor and recipient procedures; three (14.3%) centres use the same surgeon.

#### Vascular Management

**FIGURE 3:**
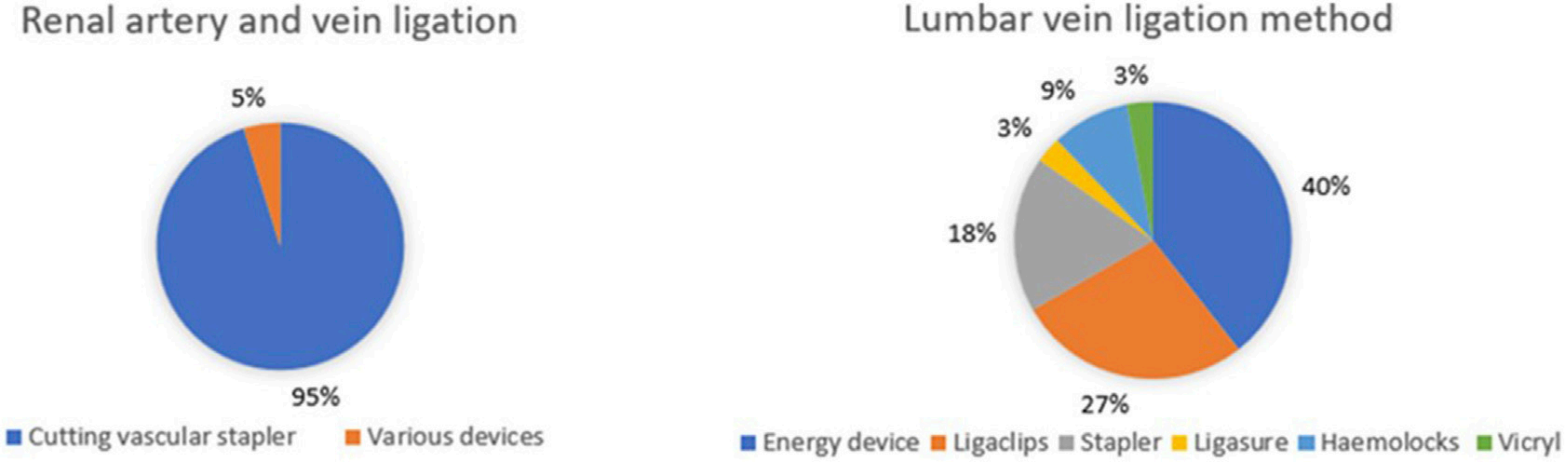
Intra-operative management of renal and lumbar vessels during donor nephrectomy.

Renal vessels are managed similarly (Figure 3): 20 (95.2%) centres use cutting vascular staplers (mostly Ethicon). None of the centres reported to use the Hemolok clips on the main renal artery. Seven centres (33.3%) use clips on smaller veins; whereas the remaining 14 (66.6%) do not use Haemolock clips at all for vessels. Six (28.6%) of the responding centres routinely administer mannitol prior to clamping of the renal vessels. Broader variation is seen in how the lumbar veins are ligated, with six different methods being used nationwide. There is also technical variation in the way the ureter is managed, with three main techniques: Hemolock clips (8 centres, 38%), Ligaclips (6 centres, 28.6%) and stapler (6 centres, 28.6%), with one centre (4.8%) using an energy device.

#### Organ Storage and Perfusion

Ten (47.6%) centres bag and box the kidney whereas nine (42.9%) centres store it on ice. The remaining two centres (9.5%) use a combination of the two methods. In three centres (14.3%), a member of the donor operating team perfuses the kidney. In 12 (57.1%) centres it is exclusively carried out by another team member and six centres (28.6%) use either method.

#### Kidney perfusion Following Nephrectomy

Perfusion fluid varies centre to centre. Seven centres (33.3%) currently use Custodial fluid, six centres (28.6%) use Histidine-Tryptophan-Ketoglutarate (HTK), three (14.3%) Servator B, two (9.5%) University of Wisconsin solution (UW), one (4.8%) UK, one (4.8%) Hyperosmolar citrate (Soltran) and one (4.8%) Celsior. This was affected by the period of the survey with centres reporting changes in the preferred fluid depending on national availability. Most centres run the fluid until it is clear, with five (23.8%) units perfusing a minimum of 1 L even if already clear. Five centres (23.8%) use unfractionated heparin in the fluid and 16 (76.2%) do not.

#### Anaesthetic Technique

Inhalational anaesthesia is used for maintenance in 12 (57.1%) centres; total intravenous anaesthesia (TIVA) in four (19%); and a combination of techniques used in five (23.8%). Arterial line and cardiac output monitoring are routinely utilised in two (9.5%) centres. Compound sodium lactate was the preferred IV maintenance fluid (61.9%), followed by saline (23.8%) and Plasmalyte (14.3%).

**FIGURE 4:**
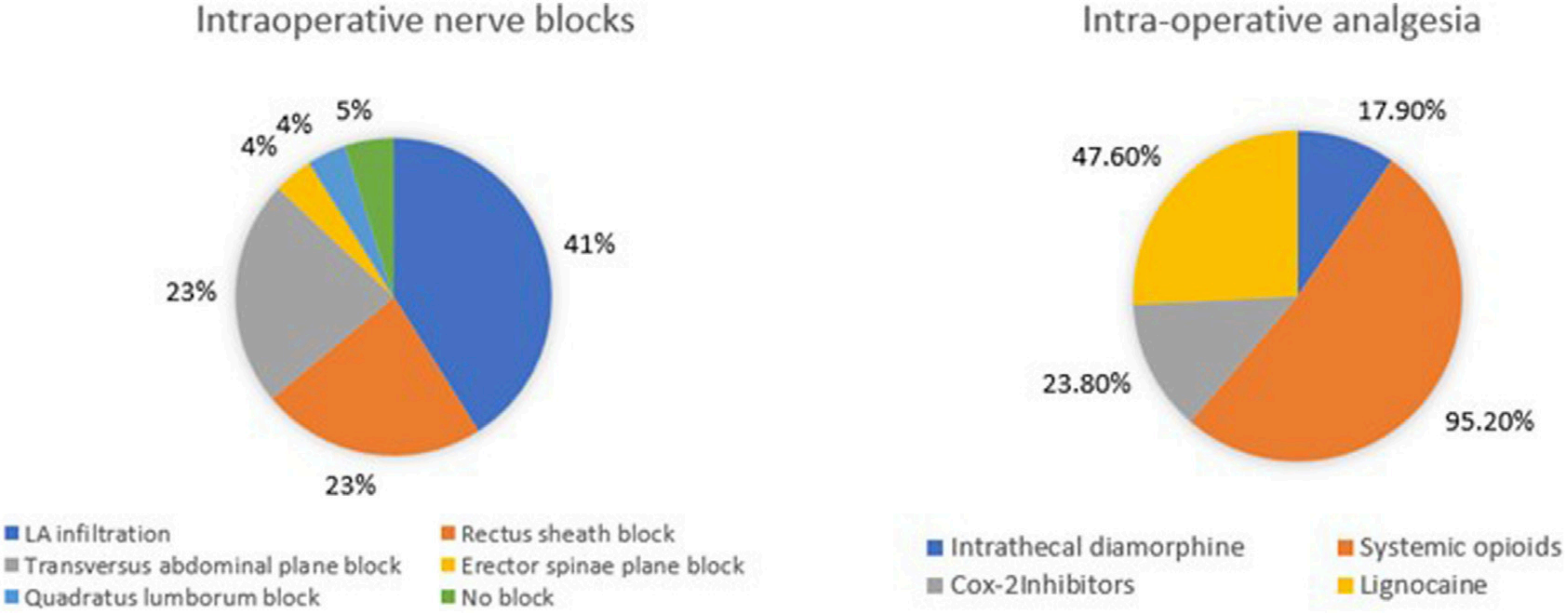
Intra-operative analgesic techniques utilised for donor nephrectomy.

Spinal anaesthesia with intrathecal diamorphine as part of multimodal analgesia is used by 17 (81%) centres. Figure 4 illustrates the diverse range of nerve blocks and opioid analgesics utilised with 10 (47.6%) of 21 units routinely performing local anaesthetic (LA) infiltration at the wound/port sites. Five (23.8%) administer cyclo-oxygenase (COX) 2 inhibitors intra-operatively.

### Postoperative Management and Follow-Up

**FIGURE 5:**
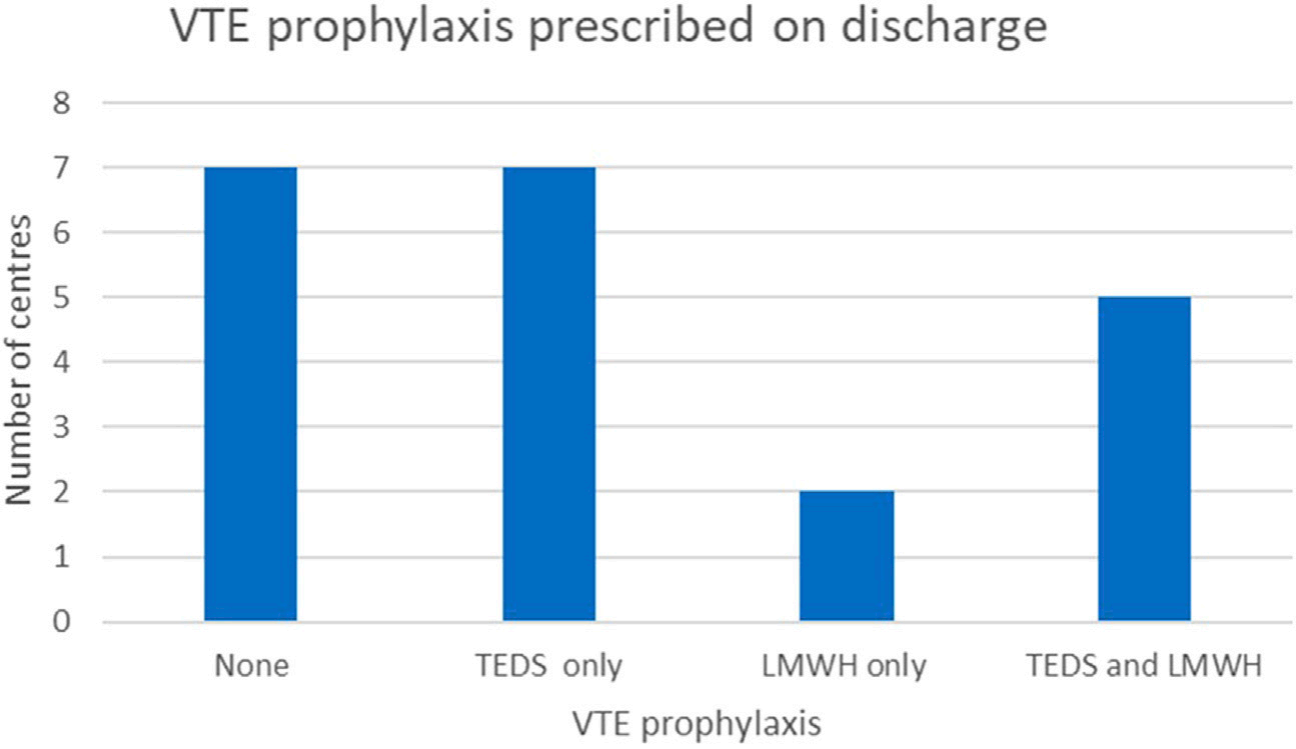
Postoperative venous thrombo-embolism (VTE) prophylaxis strategies utilised across UK transplant centres.

ERAS protocols are implemented in 13 (61.9%) centres; the same number also co-manage both donors and recipients within a single ward. There is variability in the timing of urinary catheter removal, choice of post-operative patient-controlled analgesia (PCA) opioid, and VTE prophylaxis following discharge. It is noteworthy that seven centres (33.3%) do not offer routine VTE prophylaxis at discharge, instead opting to provide mobility advice (Figure 5).

The duration of follow-up also varies, ranging from 3 months to lifelong: 12 (57.1%) centers offer lifelong care; 8 restrict it to a period of 3–12 months; and one (4.8%) provides care for 2 years.

## Discussion

This national survey reveals significant variation in the pre-operative, intra-operative, and post-operative elements of LDKT, with Table 1 displaying some of these results.

**TABLE 1:** Per centre summary table including expedited pathway availability, follow-up duration, eligibility cut-offs and ERAS implantation.

Centre	High vs. low volume centre (<40 or 40 and above)- adult LDKT performed in 23/24	Pre-op visits required	Pre-op pathway <18 weeks	Pre-op pathway <10 weeks	Follow up duration	Follow up details if provided
Regional Nephrology and Transplant Unit, Belfast City Hospital, Belfast Health and Social Care Trust, Belfast BT9 7AB, UK	High	1	Yes	Yes	Life long	
Queen Elizabeth Hospital Birmingham, University Hospitals Birmingham NHS Foundation Trust, Birmingham B15 2TH, UK	High	4	No	No	Within first 3 months	
NHS Blood and Transplant, Stoke Gifford, Bristol, UK	High	3	No	No	Life long	6 weeks, 6 months then annually
Addenbrookes hospital Addenbrooke’s, Hills Road, Cambridge, CB2 0QQ	Low	2	Yes	Yes	Life long	
Cardiff Transplant Unit, University Hospital of Wales, Cardiff and Vale University Health Board, Cardiff CF14 4XW, UK	High	4	Yes	Yes	Life long	
University Hospital Coventry, University Hospitals Coventry and Warwickshire NHS Trust, Coventry CV2 2DX, UK	Low	7	Yes	No	Within 3 months	
Edinburgh Transplant Unit, Royal Infirmary of Edinburgh, NHS Lothian, Edinburgh EH16 4SA, UK	High	2	Yes	Yes	Life long	
West of Scotland Kidney Transplant Unit, Queen Elizabeth University Hospital, NHS Greater Glasgow and Clyde, Glasgow G51 4TF, UK	High	5	Yes	Yes	Within a year	
St James' University Hospital, Leeds Teaching Hospitals NHS Trust, Leeds LS9 7TF, UK	Low	3 or 4	Yes	No	Life long	6–8 weeks post-op initial f/u with donor surgeon, then lifelong follow up
Leicester General Hospital, University Hospitals of Leicester NHS Trust, Gwendolen Road, Leicester LE5 4 PW, UK	Low	4	Yes	No	Life long	2 weeks, 3 months, then yearly
Renal and Transplant Centre, Royal Liverpool University Hospital, Liverpool University Hospitals NHS Foundation Trust, Prescot Street, Liverpool L7 8XP, UK	Low	2	Yes	No	within first 3 months	
Manchester Centre for Transplantation, Manchester Royal Infirmary, Manchester University NHS Foundation Trust, Oxford Road, Manchester M13 9WL, UK	High	3	No	No	2 years	Telephone follow up at 2 days by coordinator, telephone by surgeon at 6 weeks. Bloods at 4 weeks. Annual review for 2 years by coordinators and subsequently by GP
Freeman Hospital, Newcastle upon Tyne Hospitals NHS Foundation Trust, Newcastle upon Tyne NE7 7DN, UK	High	5	No	No	Life long	
Renal and Transplant Unit, Queens Medical Centre, Nottingham University Hospitals NHS Trust, Nottingham NG7 2UH, UK	Low	4	Yes	No	Life long	
Oxford Transplant Centre, Churchill Hospital, Oxford University Hospitals NHS Foundation Trust, Oxford OX3 7LE, UK	High	2	Yes	Yes	Life long	6 weeks post op then annually
Southwest Transplant Centre, Derriford Hospital, University Hospitals Plymouth NHS Trust, Plymouth PL6 8DH, UK	Low	2	Yes	No	Within first 3 months	
Wessex Kidney Centre, Queen Alexandra Hospital, Portsmouth Hospitals University NHS Trust, Portsmouth PO6 3LY, UK	Low	7	Yes	No	Within first 6 months	
Department of Renal Transplantation, Northern General Hospital, Sheffield Teaching Hospitals NHS Foundation Trust, Sheffield S5 7AU, UK	Low	2	Yes	Yes	Life long	
Department of Transplantation and Dialysis Access Surgery, St Georges Hospital, St Georges University Hospitals NHS Foundation Trust, London SW17 0QT, UK	High	3	No	No	Life long	2, 6, 12 weeks then annually
Department of Nephrology, Urology and Renal Transplantation, Royal Free Hospital, Royal Free London NHS Foundation Trust, London NW3 2QG, UK	Low	7	Yes	No	Within first 3 months	
Royal London Hospital Transplant Department, The Royal London Hospital, Barts Health NHS Trust, Whitechapel Road, London E1 1FR, UK	Low	minimum of 4	Yes	No	Within first 3 months	

Follow up conducted by	Eligibility BMI (min/max)	Eligibility age (min/max)	Patients accepted on one antihypertensive?	Patients accepted on dual antihypertensives?	ERAS?	Standard operation
Surgeon, transplant coordinator	35	23–85	Y	Y	Yes	Fully transperitoneal laparoscopic
Surgeon	32	18–75	Y	Y	Yes	Hand assisted transperitoneal
Surgeon, then Nephrologist	18–35	18–80	Y	Y	Yes	Fully transperitoneal laparoscopic
Surgeon and transplant coordinator	18–35	No limit	Y	Y	Yes	Fully transperitoneal laparoscopic
Nephrologist	30	23–79	Y	Y	Yes	Hand assisted transperitoneal
Surgeon, nephrologist, transplant coordinator	32	18- no limits	Y	Y	No	Fully transperitoneal laparoscopic
Surgeon	30	18-no limit	Y	Y	Yes	Hand assisted transperitoneal
Donor coordinator	No limit	21–80	Y	N	Yes	Hand assisted transperitoneal
Surgeon and transplant coordinator	30	No limit	Y	Y	Yes	Fully transperitoneal laparoscopic
Surgeons and transplant coordinator	33	18–85	Y	Y	No	Hand assissted retroperitoneal
surgeon	28	18–70	Y	N	No	Fully retroperitoneal
Surgeons	35	25–86	Y	Y	Yes	Hand asissted transperitoneal laparoscopic
surgeon then transplant coordinator	30	21-no limit	Y	Y	Yes	Fully transperitoneal lapacroscopic
surgeons and transplant coordiantor	30	25–75	Y	N	No	Fully transperitoneal and hand-assissted transperitoneal
Living donor and MDT coordiantor	35	18–82	Y	Y	No	Full transperitoneal laparoscopic
Surgeon	18–32	18–75	Y	Y	No	Hand assissted transperitoneal
Surgeon	30	18–85	Y	N	Yes	Hand asissted transperitoneal laparoscopic
Surgeon first year then transplant coordinator	30	18–70	Y	N	No	Hand assisted transperitoneal
Surgeon, transplant coordinator, nephrologist	17–32	18–75	Y	Y	No	Hand-assissted transperitoneal
Surgeon and trnasplant coordinator	17–33	18–35	Y	N	Yes	Fully retroperitoneal and hand assissted laparoscopic
Surgeon, neprhologist and trnasplant coordinator	No limit	No limit	Y	Y	Yes	Hand assisted transperitoneal and hand assisted retroperitoneal

It underscores essential opportunities to enhance and streamline the LDKT process, ultimately fostering increased donor participation and improved patient experiences. The findings of this national survey contribute to the aims of the Organ Donation and Transplantation 2030 strategy by establishing a benchmark for current practice across UK transplant centres [5]. The Kidney Care UK Transplant report 2024 identified unacceptable discrepancies in the care provided to individuals [10] and emphasises the variation among units, akin to our study, regarding the likelihood of a person receiving a living donation or being placed on a waitlist prior to starting dialysis. It also revealed differences in the workup and listing processes.

The survey demonstrates that LDKT practice across the UK is highly heterogeneous. The British Transplantation Society (BTS) guidelines [11] recommend that donor assessments be structured to minimise inconvenience and incorporate flexibility regarding timelines, consultations, investigations, and surgery scheduling. Despite this, only 50% of centres meet the recommended 18-week evaluation timeframe, whilst just two (9.5%) centres offer expedited workups under 6 weeks. These fast-track pathways represent models of good practice and could be considered for wider adoption, particularly in more pressing or pre-emptive transplant scenarios. Although pre-emptive transplantation is widely recognised as the optimal scenario for recipient outcomes [1, 2], this survey did not collect centre-specific or national proportions of pre-emptive LDKT. Consequently, we could not assess whether expedited donor pathways increase pre-emptive transplantation rates. Future national data collection should link evaluation efficiency with transplant timing to determine whether accelerated—but safe—donor preparation enables more recipients to avoid dialysis.

Variation in the number and structure of pre-operative clinic appointments points to potential inefficiencies. Centres offering combined clinics—including integrated MDT and Independent Assessment appear best placed to minimise patient burden and accelerate the pathway without compromising safety. These expedited pathways represent an opportunity for broader national adoption, potentially enhancing the donor experience, streamlining donor care and improving accessibility to transplantation. In the USA, it has been shown that donor evaluation may be too long and that long duration can lead to missed opportunities for LDKT [12]. Streamlined clinics should be tailored to patient needs with opportunities to slow down the process if required.

Risk communication exhibits variability across different centres. Although all centres address the risks associated with surgery and anaesthesia, the statistics related to donor mortality and renal failure are presented through a diverse array of figures. A recent study by Massie et al. estimates donor mortality at 3 in 10,000 (or 1 in 3333), which is regarded as the most precise statistic when evaluating LDKT from 1994 to 2009 [13]. Furthermore, the systematic review by Kortram et al. [14] highlights the necessity for guidelines that facilitate the provision of information and the acquisition of informed consent to adequately prepare prospective donors. While this survey captured quantitative risk figures, it did not capture *how* risks are conveyed—who provides counselling, whether decision aids or written materials are used, or if a reflection or “cooling-off” period is offered. We have acknowledged this omission as a limitation and recommend national adoption of evidence-based communication tools, including validated decision aids, short educational videos, standardised written leaflets, and teach-back techniques. Involving donor advocates and documenting comprehension checks would further align consent processes with the *Montgomery* principles [15].

Variation in donor eligibility criteria, particularly around age, BMI, and hypertension, suggests an opportunity for greater national collaboration. For instance, centres with more permissive criteria could accept donors referred from stricter centres, increasing the overall donor pool and reducing transplant waiting times. With obesity rates rising, flexible inter-centre referrals for donors outside local BMI thresholds could substantially benefit national transplant activity but is important to recognise that obesity is a factor that could also affect long term risk for kidney failure. Additionally, nearly all centres accept Jehovah’s Witness donors, demonstrating an encouraging trend toward inclusivity.

Preoperative imaging, protocols, and admission practices exhibit notable differences. While most centres prefer CT angiography and the retrieval of the left kidney, the decisions made by individual centres are often nuanced. Admission practices also show significant variation, with approximately half of the centres admitting donors the day prior to surgery. Although admitting patients on the day of surgery could enhance convenience for the patient and decrease hospital bed occupancy, practical constraints, especially for donors involved in paired or pooled exchanges, must be considered. The impact of preoperative intravenous fluid administration on the day of admission remains unclear.

Technically, all centres offer laparoscopic nephrectomy, however laparoscopic surgical techniques are diverse and encompass five different laparoscopic approaches. Hand assisted transperitoneal, hand assisted retroperitoneal, fully retroperitoneal, fully transperitoneal and robotic transperitoneal. Given the anatomical variation in donors, wider adoption of multiple techniques in a centre may benefit patient outcomes and broaden surgeon experience. However, this is entirely dependent on centre volume and linked to training and mentoring opportunities. This area forms a fertile area for national collaboration for patient benefit. Further exploration of technique-specific benefits could optimise patient outcomes and inform surgeon training. Only three (14.3%) centres reported to have a dedicated living donor surgical fellow/trainee in the department. This is a rich training resource, and more dedicated national living donor nephrectomy surgical fellowships should exist. No centres use the Hemolok clips on the main renal artery which is consistent to advice provided by the FDA [16]. Perfusion fluid usage prior to implantation varies widely, with seven different fluids in use. While centres report changes based on national availability, this inconsistency may affect graft outcomes and warrants further exploration or national procurement guidance.

Anaesthetic protocols also show wide variability. While most centres prefer inhalational anaesthesia for maintenance, some centres employ a TIVA technique. This may be due to a better recovery profile of TIVA [17]. Intrathecal diamorphine and local infiltration techniques remain the most common regional analgesic technique utilised intra-operatively. Though spinal anaesthesia was utilised for intraoperative pain by multiple centres, the study by Bhatia et al, failed to show any significant differences in donor outcomes, when it was compared with the surgically performed rectus sheath block for hand-assisted donor nephrectomy [18]. The evidence of good analgesia after intrathecal diamorphine in doses >200 μg was reported to be very low in one meta-analysis [19]. Quadratus lumborum block was being utilised in nearly 40% of centres as per this survey but it was not found to be superior to standard multimodal analgesia technique in a recent study [20]. Standardising anaesthetic care, where evidence supports improved recovery or outcomes, may further support ERAS protocols and enhance the donor experience and fast track recovery.

Intra-operatively, compound sodium lactate s was the preferred crystalloid for fluid maintenance in majority of centres followed by 0.9% normal saline and Plasmalyte. Recent randomised trial by Collins et al. [21] suggested that a balanced crystalloid solution should be utilised as the standard IV fluid for deceased kidney transplantation. The implications of using 0.9% saline on donors following LDKT, warrants further research given its association with hyperchloraemic metabolic acidosis.

Cross-matched blood is routinely available in theatre for LDKT in 38.1% of centres. Blood transfusion rates of <1% have been reported in LDKT [22, 23] and the use of minimal invasive techniques have further contributed to lower blood loss during donor nephrectomy. The maximum surgical blood order for LDKT should be a group and save (type and screen) sample because of the high crossmatch to transfusion ratio. This presents a potential opportunity for cost-saving in this cohort.

Post-operatively, 62% of the centres manage donors and recipients on the same ward. Variation exists in the length of catheterisation, analgesia, and mobilisation strategies. Of note, seven centres offered no routine VTE prophylaxis at discharge, relying on mobility advice alone. While rare, donor mortality due to pulmonary embolism has been reported and underscores the need for further research and consensus on postoperative VTE prophylaxis. Follow-up practices are equally diverse, with only half of centres offering lifelong follow-up as recommended by BTS guidelines [8]. Standardising long-term care is essential to ensuring ongoing donor safety and identifying late complications.

Future research is needed to link variation in practice to clinical outcomes such as donor complications, graft function, and donor satisfaction to identify which practices offer the best results. This study highlights the benefit of further research in the investigation of Shared Eligibility Models. Specifically to explore inter-centre referral models for donors who fall outside individual centre thresholds (e.g., age or BMI) and evaluate their feasibility, safety, and acceptability.

In summary, this national survey demonstrates the diversity of LDKT practice across the UK, with marked variability in all phases of care. There is no evidence from the national data that this has led to a variation in outcomes. However, while clinical outcomes remain excellent, targeted standardisation of key aspects—risk communication, eligibility criteria, surgical techniques, perioperative protocols, and follow-up could streamline the donor journey, improve experience and safety, and ultimately support the national objective to increase LDKT, particularly pre-emptive transplants.

## Strengths and Limitations

The strengths of the survey include a high response rate ensuring a broadly representative sample of UK transplant centres. The 65-question survey with contributions from a range of professionals involved in LDKT, covered all aspects of the donor pathway, providing the UKs first holistic overview of practice variation. The findings align with and contribute to the goals outlined in the UKs ‘Organ Donation and Transplantation 2030’ strategy, providing areas for improvement. The study not only identified variation but also highlighted examples of good practice, offering models that other centres could adopt.

However, several limitations should be acknowledged. First, the survey relied on centre-reported data, which may be subject to recall or social-desirability bias. Second, it captured routine practice but not evaluated patient outcomes, precluding inference on clinical effectiveness. Third, specific data on the proportion of pre-emptive LDKTs, the content of donor follow-up, and the methods of risk communication were not collected. These omissions have been explicitly stated, and corresponding recommendations are included in the Discussion. Fourth, the 12-month data collection period during which the survey was performed overlapped with changes in national supply and practice; thus, temporal bias cannot be excluded. Finally, descriptive analyses were used, and no inferential testing was performed.

## Conclusions

Living donor nephrectomy (LDN) is a unique and ethically complex surgical procedure in which a healthy individual donates a kidney to benefit a recipient with kidney failure. It remains the treatment of choice for many patients with end-stage kidney disease and has been actively promoted over the past 50 years, both globally and within the UK. While historically dominated by related and directed donations, the living donation landscape has evolved significantly in the last decade.

This national survey, capturing data from 21 of 23 UK transplant centres, reveals considerable variation in practice of management of LDN. The findings highlight a clear opportunity for greater national alignment in key areas of the LDKT pathway. While many suggested improvements may seem incremental, applying the principle of “aggregation of marginal gains” could have a meaningful cumulative impact on donor experience and pathway efficiency. This, in turn, may help increase the number of LDN performed in the UK and provides a strong foundation for further collaborative discussion. By addressing variation and promoting best practices, the quality and consistency of donor care can be improved. Addressing research gaps identified in this study are recommended to drive continued improvement in living donor transplantation across the UK.

## Data Availability

The original contributions presented in the study are included in the article/Supplementary Materials. Further enquiries can be directed to the corresponding author.
