# Supplementary material for: The Variation in Practice of the Living Donor Kidney Transplant Pathway in the UK: Results of a National Survey
Source: Transpl Int. 2025 Nov 12;38:15341. doi: 10.3389/ti.2025.15341 (PMC12650412; doi:10.3389/ti.2025.15341)
Supplement: Supplementary file 1 [file DataSheet1.pdf]

Live donor nephrectomy

Thank you for taking part in this survey which seeks to capture the pathway of living donor assessment, the different techniques of nephrectomy and follow up of donors.

The survey was conceived with the premise that there are variations in practice among different centres and we hope to capture these variations that lead to the excellent national outcomes in the annual NHSBT living donor transplant reports. A significant part of the survey focuses on anaesthetic management and post-operative pain relief. We hope to present the outcomes of this survey at local and national renal and transplant forums, for dissemination and publication.

The questions have been generated with input from several clinicians from different centres and by Lisa Burnapp (AMD Living Donation and Transplantation NHSBT) and the UK Living Donor Network. There are 48 questions which span the whole pathway and hence completion of the survey will require input from the different members of the MDT. Most importantly living donor coordinators, with nephrology, anaesthetic and surgical input.

One form should be completed for each centre. We envisage it will take 1-2 hours to complete the questions.
Progress should be saved if the survey form is accessed through the same link so please do liaise with colleagues prior to and during completion.

Please do get in touch with me if you do require any specific clarifications.

Thank you
Katie Nightingale, Core Surgical Trainee Manchester
Katie.nightingale@doctors.org.uk

1. 1. Transplant Centre   Dropdown

Mark only one oval.

- Belfast
- Birmingham
- Bristol
- Cambridge
- Cardiff
- Coventry
- Edinburgh
- Glasgow
- Guys St Thomas
- Leeds
- Leicester
- Liverpool
- Manchester
- Newcastle
- Nottingham
- Oxford
- Plymouth
- Portsmouth
- Sheffield
- St George’s
- The Royal Free
- The Royal London
- West London Transplant Centre (Hammersmith)

2. 2. How many non-transplanting centres refer to your transplant centre?

____

3. 3. How many weeks (on average) from the first patient discussion with a living donor coordinator does it take until the patient is ready for donation?

____

4. 4. In your centre, how many visits are required to complete a routine donor assessment (without additional specialist referral)?

____

5. 5. Does your centre have any of the following combined clinics?

Tick all that apply.

- [ ] MDT living donor clinic
- [ ] Combined surgical and nephrology clinic
- [ ] Clinic involving IA assessment
- [ ] Separate surgical and nephrology clinics

6. 6. Which of the following donor tests to assess renal function are routinely performed?

Please tick all routinely performed

Tick all that apply.

- [ ] Isotope GFR
- [ ] DMSA split
- [ ] Diuretic renogram
- [ ] CT Renal volume assessment
- [ ] Other: _______________________________

7. 7. What is the accepted age range for donor nephrectomy in your unit?

____

8. 8. What is the accepted BMI range for donor nephrectomy in your unit?

____

9. 9. Does your centre perform either of the following?

Tick all that apply.

- [ ] ABO incompatible transplants
- [ ] Antibody incompatible transplants
- [ ] None

10. 10. Are all donors discussed at a Multi Disciplinary Team meeting (MDT)?

Please answer Yes/No and if 'No' detail which patients are discussed

____

11. 11. During the consent procedure, what figure is quoted when discussing the risk of mortality ?

____

12. 12. During the consent procedure, what figure is quoted when discussing failure of the remaining kidney?

____

13. 13. What imaging techniques are utilised for donor nephrectomy in your unit?
Please tick all that apply

Tick all that apply.

- [ ] CT angio
- [ ] 3D reconstruction
- [ ] MR angiogram
- [ ] All images are discussed in the MDT

14. 14. With regard to laterality, for donation, how is a decision made at your centre?

Mark only one oval.

- ( ) Always right sided
- ( ) Always left sided
- ( ) Decision made on split function
- ( ) Decision made on arterial anatomy
- ( ) Both split function and arterial anatomy
- ( ) Other: ______________________

15. 15. When do you admit living donors to your centre prior to nephrectomy?

Tick all that apply.

- [ ] Day prior to surgery
- [ ] Day of surgery
- [ ] Other: ______________________

16. 16. Which of the following interventions are routinely undertaken in the unit immediately prior to surgery in individuals having a donor nephrectomy?

Tick all that apply.

- [ ] Thromboprophylaxis
- [ ] IV fluids
- [ ] G&S
- [ ] Cross match
- [ ] Carbohydrate oral drink
- [ ] Pre-operative analgesic loading
- [ ] Other: ______________________

17. 17. Who is involved in pre-assessment?

Tick all that apply.

- [ ] Anaesthetist
- [ ] Specialist nurse
- [ ] Consultant Surgeon
- [ ] Surgical trainee

18. 18. Is spinal anaesthesia with intrathecal diamorphine utilised for intra-operative pain relief?

Mark only one oval.

- ( ) Yes
- ( ) No

19. 19. What general anaesthetic technique (TIVA or inhalational) is preferred for the maintenance of anaesthesia in your centre?

____

20. 20. What intraoperative IV fluid is administered during the perioperative period?

Mark only one oval.

- Hartmanns
- 0.9% Normal Saline
- Plasma-lyte
- Other: ______________________

21. 21. Is arterial line routinely inserted for invasive blood pressure monitoring?

Mark only one oval.

- Yes
- No

22. 22. Is cardiac output monitoring routinely undertaken for individuals having a donor nephrectomy?

Mark only one oval.

- No
- Yes with Oesphageal doppler
- Yes with LIDCO
- Yes with Flowtrac
- Yes with non-invasive techniques (e.g. Finapress)
- Yes with Bioimpedance
- Other: ______________________

23. 23. What opiods are used for intra-operative pain relief?

Please select all that apply

Mark only one oval.

- Fentanyl
- Alfentanil
- Remifentanil
- Morphine
- Oxycodone
- Other: ________________

24. 24. Are COX-2 inhibitors (or NSAIDS) utilised for intraoperative pain relief?

Please answer Yes/No and if 'Yes' please specify

____

25. 25. Do the anaesthetists use IV local anaesthetic (e.g. lignocaine) routinely intraoperatively?

Please answer Yes/No and if 'Yes' please specify

____

26. 26. Are any of the following nerve blocks performed in your centre?

Mark only one oval.

- Rectus sheath block
- Transversus abdominal plane block
- Erector spinae block
- Quadratus lumborum block
- Local anaesthetic infiltration
- Other: ________________

27. 27. Post-operatively are both adult kidney donors and adult recipients managed in the same ward?

Mark only one oval.

- Yes, both HDU
- Yes, both ward
- No- on separate wards

28. 28. For post-operative pain relief is patient-controlled analgesia (PCA) utilised for all donors?

Mark only one oval.

- Yes
- No

29. 29. Please specify which opioid is used for PCA

Tick all that apply.

- [ ] Fentanyl
- [ ] Morphine
- [ ] Oxycodone
- [ ] Other: ______________________

30. 30. Postoperatively, are local anaesthic (LA) wound infusion catheters utilised for pain relief?

Mark only one oval.

- Yes
- No

31. 31. Which LA is utilised for wound infusion catheters?

Mark only one oval.

- Ropivacaine
- Bupivacaine
- Other: ______________________

32. 32. Which of the following is a standard operative technique in your centre?

Tick all that apply.

- [ ] Fully transperitoneal laprascopic
- [ ] Hand assisted transperitoneal laproscopic
- [ ] Fully retroperitioneal laproscopic
- [ ] Hand assisted retroperitoneal laproscopic
- [ ] Robotic transperitoneal
- [ ] Open nephrectomy
- [ ] Other: ______________________

33. 33. Operative technique

Please select all of which are performed (may be more than one per centre)

Tick all that apply.

- [ ] Sequential same surgeon
- [ ] Sequential different surgeon
- [ ] Parallel

34. 34. Which of the following are routinely administered to donors before clamping of the renal vessels?

Mark only one oval.

- Mannitol
- IV unfractionated heparin
- None

35. 35. Which device is used for arterial ligation?

Mark only one oval.

- Cutting vascular stapler
- Non cutting vascular stapler
- Other- please specify
- Other: ______________________

36. 36. What device is used for renal vein ligation?

Mark only one oval.

- Cutting vascular stapler
- Non cutting vascular stapler

37. 37. How are lumbar veins managed?

Tick all that apply.

- [ ] With an energy device
- [ ] With ligaclips
- [ ] With a stapler
- [ ] Other: ______________________

38. 38. How is the ureter managed intraoperatively?

Mark only one oval.

- With a stapler
- With an energy device
- With ligaclips
- Other: ______________________

39. 39. Which incision is used for kidney extraction?

Mark only one oval.

- Abdominal supra-umbilical
- Iliac fossa incision
- Pfanensteil incision
- Other: ____________

40. 40. How is the organ stored before implantation?

Mark only one oval.

- Bagged and boxed
- Kept in a tray
- Other: ____________

41. 41. Who perfuses the kidney?

Mark only one oval.

- Member of the operating team
- Another team member
- A mix of the above

42. 42. Does your unit have an agreed enhanced recovery after surgery (ERAS) protocol for donors?

Tick all that apply.

- [ ] Yes
- [ ] No

43. 43. When is the urinary catheter normally removed in your unit?

Tick all that apply.

- [ ] Prior to extubation
- [ ] In recovery
- [ ] Day 1 post-op
- [ ] Day 2 post-op
- [ ] On discharge
- [ ] No standard time
- [ ] Routine catheterisation not performed

44. 44. Please specify if VTE prophylaxis is prescribed on discharge?

Please detail the type (TEDS, LMWH etc) and duration

____

45. 45. Which of the following PRN opioid prescriptions are administered on discharge?

Mark only one oval.

- ( ) Codeine
- ( ) Tramadol
- ( ) Oral Morphine
- ( ) None
- ( ) Other: _______________________________

46. 46. Postoperatively, are NSAIDS utilised as part of a multi-modal analgesia?

Mark only one oval.

- ( ) Yes
- ( ) No

47. 47. How are donors usually followed up in your centre?

Mark only one oval.

- Within the first 3 months
- Within the first 6 months
- Within 1 year
- No follow up routinely
- Life long follow up
- Other: ______________

48. 48. Which MDT team member reviews the donor at follow up in your centre? Please tick all that apply

Tick all that apply.

- [ ] Living Donor Coordinator
- [ ] Nephrologist
- [ ] Surgeon
- [ ] Other: ______________

49. 49. Are donors outside BTS LD Guidelines considered as donors?

____

50. 50. Are hypertensive donors on 1 anti-hypertensive agent considered?

Mark only one oval.

- Yes
- No

51. 51. Are hypertensive donors on 2 anti-hypertensive agents considered?

Mark only one oval.

- Yes
- No

52. 52. Does your centre have dedicated living donor lists?

53. 53. Is crossmatched blood available in theatre or do you only send off a group and save?

54. 54. Is donor nephrectomy a two consultant procedure or consultant and trainee? If trainee present, is the trainee a dedicated transplant trainee or GS/Urology trainee?

55. 55. Is donation and transplantation carried out by the same surgeon or different surgeons?

56. 56. Is there a dedicated living donor surgical fellow/trainee in the department?

57. 57. How are new consultants mentored in the procedure?

58. 58. Is robotic donor nephrectomy being considered in your centre?

59. 59. Does your centre accept Jehovah's Witness Donors?

60. 60. Type/Brand of vascular stapler used?

61. 61. Are Haemolok clips used for vascular management in your centre? If yes please specify how

62. 62. What perfusion fluid is used?

63. 63. Do you use a set perfusion volume or is it subjective until clear?

64. 64. Is heparin used in the perfusion fluid?

65. 65. Is the kidney perfused by a member of the operating team or another member?

This content is neither created nor endorsed by Google.

Google Forms
